# Efficacy of fosfomycin compared to second generation cephalosporin flumarin as antimicrobial prophylaxis for transrectal ultrasound-guided prostate biopsy: a single center retrospective study

**DOI:** 10.1186/s12894-023-01391-7

**Published:** 2023-12-19

**Authors:** Hee Youn Kim, Daehyun Lim, Young Hyo Choi, Je Mo Yoo, Dong Sup Lee, Seung-Ju Lee

**Affiliations:** Department of Urology, College of Medicine, St. Vincent’s Hospital, The Catholic University of Korea, 93, Jungbu-daero, Paldal-gu, Suwon-si, Gyeonggi-do, Seoul, 16247 Republic of Korea

**Keywords:** Prostatic neoplasms, Image-guided biopsy, Antibiotic prophylaxis

## Abstract

**Background:**

Fluoroquinolone has been the historic choice of antimicrobial prophylaxis for transrectal ultrasound (TRUS) guided prostate biopsy. However, increased fluoroquinolone resistance and recent restrictions of its use for antimicrobial prophylaxis has led to the emergence of alternative agents for antimicrobial prophylaxis for TRUS guided prostate biopsy including fosfomycin and cephalosporins. This study aimed to compare the efficacy of fosfomycin and a second-generation cephalosporin flumarin as alternative antimicrobials for TRUS-guided prostate biopsy in terms of the incidence of infectious complications after TRUS-guided prostate biopsy.

**Methods:**

A retrospective chart review of all patients who underwent TRUS-guided prostate biopsy between November 2009 to January 2023 was undertaken. Comparison of baseline characteristics and the incidence of infectious complications was done between those who received fosfomycin as antimicrobial prophylaxis for TRUS-guided prostate biopsy and those who received flumarin. Multivariate logistic regression analysis was conducted to identify risk factors for infectious complications after TRUS-guided prostate biopsy.

**Results:**

Of 2,900 patients identified as eligible candidates for analysis, 333 (11.5%) received fosfomycin and 2,567 (88.5%) received flumarin. The overall rate of infectious complications was approximately 3% lower in patients who received fosfomycin, although such difference did not reach statistical significance (5.7% vs. 8.6%, p = 0.074). Multivariate logistic regression analysis showed that history of operation done under general anaesthesia within six months of the biopsy (odds ratio [OR]: 2.216; 95% confidence interval [CI]: 1.042–4.713; p = 0.039) and history of prior antimicrobial use within six months (OR: 1.457; 95% CI: 1.049–2.024; p = 0.025) were significant risk factors for infectious complications after TRUS-guided prostate biopsy.

**Conclusion:**

Fosfomycin was comparable to second-generation cephalosporin flumarin in preventing infectious complications after TRUS-guided prostate biopsy. Coupled with its properties such as ease of administration, low adverse effects, low resistance rate, and low collateral damage, fosfomycin might be an attractive alternative antimicrobial prophylaxis for TRUS-guided prostate biopsy.

**Supplementary Information:**

The online version contains supplementary material available at 10.1186/s12894-023-01391-7.

## Background

Transrectal ultrasound (TRUS) guided prostate biopsy has been the gold standard for diagnosing prostate cancer for decades. Although the recent European Association of Urology (EAU) guideline recommends transperineal prostate biopsy on grounds of decreased infectious complications compared to the transrectal method [1], several practical issues hinder drastic conversion to the transperineal method [2]. Until such hurdles are overcome, TRUS-guided prostate biopsy is likely to remain the main modality for diagnosing prostate cancer for some time.

Antimicrobial prophylaxis is mandatory prior to TRUS-guided prostate biopsy to prevent infectious complications [3]. Fluoroquinolones have been the historic choice for antimicrobial prophylaxis for TRUS-guided prostate biopsy. However, infectious complications after TRUS-guided prostate biopsy have been on the rise since the previous decade. One of the main suspected reasons is increase in fluoroquinolones resistance [4]. Coupled with the suspension of fluoroquinolones for use for antimicrobial prophylaxis by the European Commission in 2019 [5], alternative strategies for antimicrobial prophylaxis have been recommended in regions where fluoroquinolones use are restricted [1] or where resistance rate for fluoroquinolones is high [6]. Although fluoroquinolones are not restricted in South Korea, South Korea has been a region with high fluoroquinolone resistance [7–9]. As such, our institution has been using second-generation cephalosporin flumarin for antimicrobial prophylaxis for TRUS-guided prostate biopsy since 2009 until 2019.

Another recently recommended antimicrobial prophylactic agent for TRUS-guided prostate biopsy is Fosfomycin [10]. Using fosfomycin as an antimicrobial prophylaxis agent for TRUS-guided prostate biopsy has several advantages such as high activity against multidrug resistant strains, low resistance rate, good safety profile, and good penetration into prostate [11]. We have been using it as our main antimicrobial prophylaxis for TRUS-guided prostate biopsy since 2019. Multiple studies have reported that it is efficacious in terms of antimicrobial prophylaxis for TRUS-guided prostate biopsy [12, 13], although opposing views also exist [14]. Most studies have compared fosfomycin with fluoroquinolones. As such, the aim of the present study was to determine the efficacy of fosfomycin compared with a second-generation cephalosporin flumarin in terms of incidence of infectious complications after TRUS-guided prostate biopsy.

## Methods

### Study population and design

A retrospective chart review was undertaken for all patients who underwent TRUS-guided prostate biopsy between November 2009 and January 2023. Patients who received either fosfomycin or flumarin as an antimicrobial prophylaxis agent before TRUS-guided prostate biopsy with at least one month of follow-up period were included for analysis. The following patients were excluded from this study: patients who received antimicrobial prophylaxis other than fosfomycin or flumarin, patients with less than one month of follow-up period after the procedure, patients who simultaneously underwent other procedures or surgeries, and patients who were admitted in other departments during the procedure. The following information were obtained: age (years), history of diabetes mellitus (DM), health care risk, history of operation done under general anaesthesia within six months of TRUS-guided prostate biopsy, history of treatment for urinary tract infection (UTI) within six months, history of prior prostate biopsy, history of prior antimicrobial use within six months, prostate specific antigen (PSA) level, prostate size, number of biopsy cores, cancer detection rate, and infectious complications rate. Health care risk was defined as history of admission due to any cause within 90 days, history of urethral catheterization within 30 days, history of invasive urologic procedures within 30 days, and history of dialysis and chemotherapy at the time of the procedure. Infectious complications were defined as one or more of the following symptoms after the biopsy procedure that led clinician to prescribe unplanned antimicrobials: frequency, urgency, dysuria, suprapubic discomfort, foul-smelling urine, scrotal pain, and fever following biopsy [15]. The need for hospitalization due to UTI was based on the assessment of the practicing clinician. The current study was approved by the Institutional Review Board of St. Vincent’s Hospital, the Catholic University of Korea (VC20RESI0046).

### Procedure

We have previously described our procedure for TRUS-guided prostate biopsy [16]. Briefly, a standard 12-core TRUS-guided prostate biopsy was performed for patients in a left lateral position. All patients underwent a rectal povidone-iodine preparation before the procedure. Enema was not done. For antimicrobial prophylaxis, flumarin was used from November 2009 to November 2019 and fosfomycin was used from December 2019 to January 2023. Flumarin 1 g was administered intravenously within one hour prior to the procedure. Fosfomycin 3 g was administered orally the night before the procedure.

### Statistical analysis

All statistical analyses were done using SPSS (IBM Corp. Released 2012. IBM SPSS Statistics for Windows, Version 21.0. Armonk, NY: IBM Corp.). Descriptive statistics were used to describe categorical variables. Mean and standard deviation were used to describe continuous variables. Categorical variables were compared with either chi-square test or Fisher’s exact test. Continuous variables were compared with Mann-Whitney U test. Multivariate logistic regression analysis was conducted to identify risk factors of infectious complications after TRUS-guided prostate biopsy. Statistical significance was considered when p-value was less than 0.05.

## Results

Comparison of baseline characteristics and infectious complications between patients who received fosfomycin and those who received flumarin for antimicrobial prophylaxis for TRUS guided prostate biopsy are described in Table 1. Of 2,970 patients who underwent TRUS-guided prostate biopsy during the study period, 2,900 patients were identified as eligible candidates for analysis. Of these patients, 333 (11.5%) received fosfomycin as an antimicrobial prophylaxis agent for TRUS-guided prostate biopsy and 2,567 (88.5%) received flumarin. There was no statistical difference in age, DM, health care risk, history of operation within six months, history of UTI within six months, or prior antimicrobial use within six months between the two groups. History of prior prostate biopsy was lower in patients who received fosfomycin (5.1% vs. 13.4%, p = 0.000). The overall rate of infectious complications was approximately 3% lower in patients who received fosfomycin, although such difference did not reach statistical significance (5.7% vs. 8.6%, p = 0.074). When patients with infectious complications were stratified into those who required hospitalization and those who did not, the rate of infectious complications decreased substantially in both groups to be only 0.9% in patients who received fosfomycin and 0.3% in patients who received flumarin. Although the rate of hospitalization due to infectious complications was three times higher in patients who received fosfomycin, the difference between the two groups did not reach statistical significance as well (p = 0.073). Information of patients with positive culture and their susceptibility patterns are provided in Supplementary Table 1.

**Table 1:** Comparison of baseline characteristics and infectious complications between patients who received fosfomycin and those who received flumarin for antimicrobial prophylaxis for TRUS guided prostate biopsy

	Fosfomycin	Flumarin	*p*
n	333	2567	
Age	68.3 ± 8.8	67.5 ± 8.7	0.161^1^
DM	54 (16.2%)	405 (15.8%)	0.836^2^
Health care risk^*^	8 (2.4%)	120 (4.7%)	0.058^2^
Operation < 6 months	6 (1.8%)	47 (1.8%)	0.970^2^
UTI < 6 months	4 (1.2%)	18 (0.7%)	0.308^3^
Prior prostate biopsy	17 (5.1%)	344 (13.4%)	0.000^2^
Prior antibiotics < 6 months	57 (17.1%)	503 (29.6%)	0.265^2^
PSA Group			0.261^2^
< 10	196 (58.9%)	1566 (61.0%)	
10–20	66 (19.8%)	547 (21.3%)	
> 20	71 (21.3%)	454 (17.7%)	
Prostate size	44.5 ± 20.9	44.9 ± 20.8	0.789^1^
No of biopsy cores	11.4 ± 1.8	10.8 ± 2.0	0.000^1^
Cancer detection	146 (43.8%)	887 (34.6%)	0.001^2^
Infectious complications (overall)	19 (5.7%)	220 (8.6%)	0.074^2^
Infectious complications (hospitalization)	3 (0.9%)	8 (0.3%)	0.073^2^

DM = diabetes mellitus, UTI = urinary tract infection, FQ = fluoroquinolone, PSA = prostate specific antigen

*Health care risk = admission within 90 days, urethral catheterization within 30 days, invasive urologic procedures within 30 days, dialysis, chemotherapy

^1^Mann Whitney U test

^2^Chi square test

^3^Fisher’s exact test

Multivariate logistic regression analysis described in Table 2 showed that history of operation done under general anaesthesia within six months of TRUS-guided prostate biopsy (odds ratio [OR]: 2.216; 95% confidence interval [CI]: 1.042–4.713; p = 0.039) and history of prior antimicrobial use within six months (OR: 1.457; 95% CI: 1.049–2.024; p = 0.025) were identified as significant risk factors for infectious complications after TRUS-guided prostate biopsy. The type of antimicrobial agents (fosfomycin or flumarin) was found to be unrelated to the occurrence of infectious complications after TRUS-guided prostate biopsy.

**Table 2:** Multivariate logistic regression to identify risk factors of infectious complications after TRUS-guided prostate biopsy

Variables	OR (95% CI)	P value
Age	0.951 (0.938–0.965)	0.000
Operation < 6 months	2.216 (1.042–4.713)	0.039
UTI < 6 months	2.417 (0.839–6.961)	0.102
Prior antibiotics < 6 months	1.457 (1.049–2.024)	0.025
Antibiotics used	1.494 (0.916–2.438)	0.444

## Discussion

The current study aimed to identify the efficacy of fosfomycin compared with flumarin in terms of the incidence of infectious complications after TRUS-guided prostate biopsy. As most clinical trials studying the efficacy of fosfomycin as antimicrobial prophylaxis for TRUS-guided prostate biopsy compared fosfomycin with fluoroquinolones, we conducted this study to compare fosfomycin with a second-generation cephalosporin. To the best of our knowledge, this has not been done previously. Both were not commonly used as antimicrobial prophylaxis agents in TRUS-guided prostate biopsy as fluoroquinolones have been the main recommended choice of antimicrobial prophylaxis agents for TRUS-guided prostate biopsy for decades. However, the increase in infectious complications after TRUS-guided prostate biopsy mainly due to increased fluoroquinolone resistance [4] and restricted use of fluoroquinolones by the European Commission in 2019 [5] has prompted the guidelines to recommend alternative strategies for antimicrobial prophylaxis for TRUS-guided prostate biopsy. This includes targeted prophylaxis based on rectal swab, augmented prophylaxis, and alternative antibiotics like fosfomycin and cephalosporins, both of which were used in our series [1].

One of the difficulties when selecting an optimal agent for antimicrobial prophylaxis is the geographical variation of antimicrobial resistance. Knowledge of local antimicrobial resistance pattern is therefore paramount in this selection process [17, 18]. Our hospital had been using flumarin as our main antimicrobial prophylaxis agent for TRUS-guided prostate biopsy since 2009 until 2019 when we switched to fosfomycin. Although there is no restriction in South Korea with the use of fluoroquinolones like the one implemented in Europe in 2019, reported fluoroquinolone resistance rate has been high. Antimicrobial susceptibility of *E. coli* to ciprofloxacin in South Korea was 84.8% in a study from 2003 [19]. This figure had decreased progressively over time; the susceptibility rate was 76.6% in a study from 2008 [7], 74.6% in a study from 2011 [8], and 69.8% in a study from 2013 [9]. Using fluoroquinolones, as recommended by the guidelines at the time, might have caused detrimental effects on patients undergoing TRUS-guided prostate biopsy. One report has suggested that if local resistance of *E. coli* to fluoroquinolones is greater than 20%, alternative antibiotics should be considered [6]. Resistance rates to cephalosporins were all under 20% in studies mentioned above. As such, we have been using second-generation cephalosporin flumarin as our main antimicrobial prophylaxis for TRUS-guided biopsy for more than 10 years.

The addition of fosfomycin as one of the recommended alternative antimicrobials for TRUS-guided prostate biopsy in guidelines took place in relatively recent years, although the drug itself was first developed in 1969. Its favorable properties such as high activities against multidrug resistant strains, low resistance rate, good safety profile, good penetration into prostate, and so on [11] have facilitated its addition, especially in the era of increased fluoroquinolone resistance and restrictions of fluoroquinolone use. Another important advantage is that there is no cross-resistance or parallel resistance against fosfomycin, meaning that fosfomycin exerts less collateral damage on the microbiome than other broad-spectrum antimicrobials such as fluoroquinolones and cephalosporins [13, 20]. These factors were the main reasons for switching from second-generation cephalosporin flumarin to fosfomycin as our main antimicrobial prophylaxis agent for TRUS-guided prostate biopsy in our hospital.

We found that infectious complications (total as well as those requiring hospitalization) were comparable between the two groups and that prior history of operation done under general anaesthesia and antibiotics use within six months were significant factors for infectious complications, both of which confirms findings from previous studies that showed the relationthiop between prior antibiotics exposure and risk of antibiotic resistance-related UTI [21]. The type of antimicrobial agent (fosfomycin or flumarin) was found to be unrelated to infectious complications. Considering the advantages of fosfomycin such as ease of administration, low adverse effects, low resistance rate, and low collateral damage, fosfomycin might be a attractiven alternative antimicrobial prophylaxis for TRUS-guided prostate biopsy. We feel that our finding may provide additional insight into antimicrobial prophylaxis for TRUS-guided prostate biopsy as the use of fluoroquinolone is expected to decrease further in the future.

The efficacy of fosfomycin in preventing infectious complications after TRUS-guided prostate biopsies has been studied in several randomized controlled trials (RCT) and meta-analysis with some conflicting results. Sen et al. in their RCT have compared single dose of 3 g fosfomycin with 500 mg of oral ciprofloxacin and concluded that fosfomycin is a strong alternative antibiotic prophylaxis for TRUS-guided prostate biopsy [13]. Lista et al. in their RCT have also compared two doses of 3 g fosfomycin with 10 doses of 500 mg oral ciprofloxacin and concluded that fosfomycin is as effective as ciprofloxacin [12]. A meta-analysis of three RCTs [3] and two other meta-analysis that included non-RCTs [20, 22] all significantly favored fosfomycin over quinolone-based prophylaxis. On the other hand, in a large Canadian cohort study involving 9,391 subjects, it was found that fosfomycin was not an effective alternative to ciprofloxacin [14]. The reason for such conflicting results might be due to limitations with clinical trials studying the efficacy of fosfomycin as antimicrobial prophylaxis for TRUS-guided prostate biopsy including variation in dosage (single dose versus double dose), variation in drug infusion time (night before versus just before procedure), heterogenous biopsy technique and follow-up protocol, variation in definition of infectious complications, and variation of fluoroquinolone resistance rate of study population [23]. Still, the overall result seems to be positive for using fosfomycin, although standardization of study parameters is needed to have a more robust conclusion.

The recent EAU guideline recommends performing prostate biopsy with a transperineal approach over a transrectal approach on two grounds: (1) a higher sensitivity for detection of clinically significant prostate cancer with the transperineal approach [24–26], and (2) lower incidence of infectious complications, sepsis, and readmission due to sepsis [17, 27–29]. On the other hand, the American Urological Association (AUA) guideline does not recommend a particular approach due to insufficient evidence [30]. With the advent of MRI guided prostate biopsy and increasing evidence of improved detection rate of clinically significant prostate cancer and lower infectious complications associated with transperineal prostate biopsy the transperineal approach is likely to become the main modality of prostate biopsy in the future. However, several practical issues hinder drastic conversion from transrectal to transperineal prostate biopsy. The transperineal approach requires additional resources in terms of equipment, operating space, and personnel which all can increase the cost [20]. General anaesthesia is usually needed with the transperineal approach [2], although feasibility of local anaesthesia has been reported [31, 32]. In addition, although the risk of infectious complications is lower with the transperineal approach than with the transrectal approach, acute urinary retention is higher with the transperineal approach, leading to similar risk of hospitalization [17]. For these reasons, switching to the transperineal approach from the already familiar, office based TRUS-guided prostate biopsy might face considerable resistance from a large proportion of urologists worldwide. Thus, the transrectal approach is likely to remain the main modality for prostate cancer diagnosis for some time.

The current study has several limitations that should be mentioned. First, this was a retrospective study prone to selection bias. Second, we did not report fosfomycin resistance, as it was not included in our routine antimicrobial susceptibility tests. However, fosfomycin resistance rate remains very low with reported susceptibility rate to *E. coli* of over 99% [33, 34]. Other studies have also omitted reporting fosfomycin resistance rate for similar reason [14, 35, 36]. Lastly, our practice of administering a single dose of 3 g fosfomycin the night before the procedure differs from others who recommend the use of 3 g fosfomycin three hours before the procedure plus 3 g 24 h after the procedure [11]. However, some studies have reported that single dose regimen may be adequate [13, 37]. As previously mentioned, standardization of these parameters is necessary in future studies.

## Conclusions

In conclusion, in the current study, fosfomycin was found to be comparable to second-generation cephalosporin flumarin in preventing infectious complications after TRUS-guided prostate biopsy. Coupled with its properties such as ease of administration, low adverse effects, low resistance rate, and low collateral damage, fosfomycin might be an attractive alternative antimicrobial prophylaxis for TRUS-guided prostate biopsy. Future studies in this respect should standardize study parameters.

### Electronic supplementary material

Below is the link to the electronic supplementary material.

Supplementary Material 1: List of patients with positive culture

## Data Availability

The datasets used and/or analysed during the current study are available from the corresponding author on reasonable request.

## Abbreviations

TRUS: transrectal ultrasound
EAU: European Association of Urology
DM: diabetes mellitus
UTI: urinary tract infection
PSA: prostate specific antigen
RCT: randomized controlled trial
