# Supplementary material for: Efficacy of fosfomycin compared to second generation cephalosporin flumarin as antimicrobial prophylaxis for transrectal ultrasound-guided prostate biopsy: a single center retrospective study
Source: BMC Urol. 2023 Dec 19;23:211. doi: 10.1186/s12894-023-01391-7 (PMC10729332; doi:10.1186/s12894-023-01391-7)
Supplement: Supplementary file 1 — Supplementary Material 1: List of patients with positive culture [file 12894_2023_1391_MOESM1_ESM.docx]

Supplementary table 1. List of patients with positive culture

| No | Age | Antibiotics used | Cultured Pathogen | Specimen | Amoxicillin/clavulanate | Piperacillin/  tazobactam | Trimethoprim-sulfamethoxazole | Ciprofloxacin | Amikacin | Imipenem | ESBL |
| --- | --- | --- | --- | --- | --- | --- | --- | --- | --- | --- | --- |
| 1 | 60 | Flumarin | *Achromobacter xylosoxidans* | Urine | Not tests | Sensitive | Sensitive | Sensitive | Sensitive | Sensitive | Negative |
| 2 | 69 | Flumarin | *Klebsiella pneumoniae* | Urine | Sensitive | Sensitive | Sensitive | Sensitive | Sensitive | Sensitive | Negative |
| 3 | 68 | Flumarin | *E.coli* | Urine and blood | Sensitive | Sensitive | Sensitive | Sensitive | Sensitive | Sensitive | Negative |
| 4 | 69 | Fosfomycin | *E.coli* | Urine | Sensitive | Sensitive | Sensitive | Sensitive | Sensitive | Sensitive | Negative |
| 5 | 74 | Fosfomycin | *Klebsiella pneumoniae* | Blood | Sensitive | Sensitive | Sensitive | Sensitive | Sensitive | Sensitive | Negative |
| 6 | 63 | Fosfomycin | *E.coli* | Urine | Sensitive | Sensitive | Resistant | Resistant | Sensitive | Sensitive | Positive |
| 7 | 60 | Fosfomycin | *Klebsiella pneumoniae* | Blood | Sensitive | Sensitive | Sensitive | Sensitive | Sensitive | Sensitive | Negative |
